# Psychometric properties of ADHD screening and diagnostic tools in patients with severe substance use disorders

**DOI:** 10.1016/j.ijchp.2026.100682

**Published:** 2026-04-16

**Authors:** Norman Therribout, Lucia Romo, Thibault Hennequin, Clara Chrétienneau, Lucie Davost, Tatiana Fontaine, Frank Bellivier, Florence Vorspan, Alexandra Dereux, Emily Karsinti, Romain Icick

**Affiliations:** a Assistance Publique – Hôpitaux de Paris, Hôpital Fernand-Widal, Département Universitaire de Psychiatrie et de Médecine Addictologique, Paris, France; b Université Paris-Cité, INSERM U1144 “Therapeutic Optimization in Neuropharmacology”, Paris, France; c Laboratoire Clipsyd EA 4430, Université Paris-Nanterre, Nanterre, France; d IHM “addictionS”, Université Paris-Cité, Paris, France; e Assistance Publique – Hôpitaux de Paris, Hôpital Raymond-Poincaré, Garches, France; f CESP, U1018 INSERM UPS UVSQ, Villejuif, France; g Département d’addictologie, hôpital René Muret, APHP, UMR-S 1144, Inserm UMR-S 1144, Université Paris-Cité, Paris, France; h Centre Hospitalier des quatre villes, unité d’addictologie, 91570 Sèvres, France

**Keywords:** Attention deficit hyperactivity disorder, Substance use disorder, Evaluation, Diagnosis, DIVA

## Abstract

**Objective:**

The aim of this study was to compare the performance of several tools for diagnosing ADHD in an adult population suffering from SUD, within a multi-tool diagnostic procedure.

**Method:**

Eighty-two patients suffering from SUD and suspected of ADHD were referred to undergo the procedure. They were asked to complete two questionnaires: the Adult Self Report Scale (ASRS-6) and the Wender Utah Rating Scale (WURS-25) and three interviews: the Mini International Neuropsychiatric Interview (MINI-S), the Dual Diagnosis Screening Interview (DDSI) and the Diagnostich Interview Voor ADHD (DIVA-5). Their performance was compared to a consensus statement provided by trained psychiatrists and psychologists informed by all the assessments.

**Results:**

All the tools showed good sensitivity (0.81–0.98), but only the DIVA-5 showed good specificity (0.88). Accordingly, the negative predictive value calculated for an assumed prevalence of ADHD of 23 % was high for all tools (0.88 – 0.99) whereas only the DIVA-5 showed a good positive predictive value (0.71).

**Conclusion:**

The ASRS, the WURS, the MINI and the DDSI are thus useful screening tools among SUD patients, but the DIVA-5 seems to provide the most accurate information to ascertain ADHD diagnosis in this population. Further investigations are needed to assess the impact of prior standardized comorbidity evaluations on the accuracy of DIVA-5.

## Introduction

Substance use is involved in 1/8 annual deaths worldwide (WHO, 2023, 2024). Substance Use Disorders (SUD) represent a set of maladaptive responses to substance use, which often lead to chronic and highly-disabling conditions where the loss-of-control over substance use is the core phenomenon (Goodman, 1990). Part of the burden of SUDs has been associated to psychiatric comorbidities (Hunt et al., 2018; Lai et al., 2015; Toftdahl et al., 2016). Attention deficit hyperactivity disorder (ADHD) - a disorder characterized with persistent attention, impulse control and emotional regulation deficits (American Psychiatric Association, 2013) - is found in 23 % of adults suffering from SUD (Rohner et al., 2023; van Emmerik-van Oortmerssen et al., 2012), representing their most frequent chronic comorbidity. While diagnosing and treating ADHD in adults is a challenge in itself (Cortese et al., 2018; Lopez et al., 2018), the association with SUD is related to more severe addiction symptoms and standard treatments for ADHD lead to poorer outcomes than without SUD (Carpentier & Levin, 2017; Icick et al., 2020). These elements support the need for systematic screening for ADHD in SUD patients, as recommended by recent consensus statements (Crunelle et al., 2018; Young et al., 2023).

To date, the screening of ADHD in adults relies on efficient self-rating tools, especially the ASRS-6 (*Adult Self-Report Scale for ADHD*) and the WURS-25 (*Wender Utah Rating Scale*)(Brevik et al., 2020; Kessler et al., 2007). Even if these tools are recommended for systematic ADHD screening in SUD individuals (Crunelle et al., 2018), some studies have shown that they are poor predictors of ADHD diagnosis in SUD samples (Chiasson et al., 2012; Daigre et al., 2015; Luderer et al., 2019). Structured interviews can be helpful in the diagnosis of ADHD in adults. Although the CAADID (*Conners Adult ADHD Diagnostic Interview*) is known as the gold standard for ADHD diagnosis (Epstein et al., 2000), only one study has reported a good test-retest fidelity in SUD patients (van Emmerik-van Oortmerssen et al., 2017). Moreover, the CAADID has not been updated to DSM-5 criteria, is relatively expensive and fairly time-consuming.

The DIVA-5 (*Diagnostich Interview Voor ADHD*) is an accessible assessment tool for ADHD diagnosis in adults, designed for DSM-5 criteria (DIVA Fundation, 2010; Kooij, 2013). This interview is widely used, and its previous version demonstrated good psychometric properties for ADHD diagnosis (Pettersson et al., 2018; Ramos-Quiroga et al., 2019). Unfortunately, to date very little is known about its accuracy in SUD samples. Other more general standardized diagnostic interviews include a specific ADHD assessment module: the Mini International Neuropsychiatric Interview for DSM-5 (MINI-S) and the Dual Diagnosis Screening Interview (DDSI) (Hergueta & Weiller, 2013; Mestre-Pintó et al., 2013), but the ADHD module has not yet been validated. Although structured interviews could be helpful in diagnosing ADHD in adults, validation studies are lacking for most of the available tools, and little is known about the psychometric validity of these assessments in the context of co-occurring SUD.

The study aimed to evaluate the performance of several screening and diagnostic instruments for Attention Deficit-Hyperactivity Disorder (ADHD) when used as part of a multi-tool diagnostic approach in individuals with Substance Use Disorders (SUD). The goal was to identify which tool(s) aligned most consistently with the final expert consensus ADHD diagnosis, reached after the completion of the diagnostic process. The hypothesis was that the DIVA-5, would demonstrate the best consistency with the expert consensus diagnosis.

## Method

### Participants

Participants were unpaid French-speaking adult outpatients receiving specialized care for SUD, referred to our tertiary care center to ascertain or refute a diagnosis of ADHD.

- Inclusion criteria: >18 years old, having at least one SUD other than tobacco-related
- Exclusion criteria: inability to complete assessments due to unstable medical condition, compulsory care, current guardianship or inability to fill out the research assessments (for example due to limited literacy or no knowledge of French)

They underwent a comprehensive assessment of ADHD and psychiatric comorbidities during a dedicated procedure, taking place in the outpatient addiction setting, Fernand-Widal academic hospital. Patients were referred by their treating clinician from word-of-mouth. The study was conducted according to the tenets of the Declaration of Helsinki (World Medical Association, 2013) and the rules of the ethics committee of Paris-Nanterre University. In accordance with usual care at Paris academic hospitals, participants were informed that their data may be used for research, with the option to object. All participants signed a consent form stating they did not object to this use. No additional approval was required.

### Measures and procedure

All participants were contacted by telephone to explain the procedure and schedule the visit. Before the interview, referring clinicians had to fulfil a form detailing developmental and medical history, current state and reasons for suspecting ADHD. Participants were asked to complete self-rated tools, including: the ASRS-6 [∼2 min] (Kessler et al., 2007), the WURS-25 [∼7 min] (Brevik et al., 2020), the Alcohol Use Disorder Identification Test (AUDIT) [∼3 min] (Gache et al., 2005), the Cannabis Use Disorder Identification Scale (CUDIT) [∼3 min] (Guillem et al., 2011) and the Fagerström Test for Nicotine Dependence (FTND) [∼3 min] (Etter et al., 1999).

On arrival, participants were accompanied by a nurse to complete administrative procedures, collect vital signs and perform biological screening of recent substance use (urine drug screening, alcohol breath testing).

Assessments were conducted in one day, including structured interviews (detailed below), cognitive tasks [∼60 min], and self-reported questionnaires in a systematic order [∼60 min in total]. Full details on the assessment procedure are available in Therribout et al. (Therribout et al., 2022). Investigators were experienced clinical specialists (psychiatrists or psychologists) with substantial experience in managing ADHD.

Structured interviews include:

An anamnestic interview [∼30 min] for the main clinical and socio-demographic background, including the number of DSM-5 criteria for the main current substance use disorder (identified by asking to the participant about the substance they currently define as the most problematic).

- The Mini International Neuropsychiatric Interview Simplified for DSM-5 (MINI-S), providing a categorical diagnosis for 14 neuropsychiatric disorders including ADHD and SUDs [∼90 min]. It has been validated for the depressive symptoms module (Hergueta & Weiller, 2013) and a previous version based on DSM-IV criteria (MINI-Plus), showed acceptable validity for screening on adult ADHD in an SUD sample (Palma-Álvarez et al., 2020).
- The Dual Disorder Screening Instrument (DDSI), was used as part of the primary cohort objective of the French validation of this instrument (Mestre-Pintó et al., 2013). This interview, based on DSM-IV-TR criteria, investigates common SUD comorbidities such as panic disorder, generalized anxiety, phobias, agoraphobia, dysthymia, major depressive disorder, bipolar disorder, ADHD, Post-Traumatic Stress Disorder, and psychosis [∼45 min].
- The Diagnostic Interview for ADHD in adults (DIVA-5), one of the structured interviews recommended for the assessment of ADHD in adults suffering from SUDs (Crunelle et al., 2018) was also used to assess current and childhood ADHD symptoms (DIVA Fundation, 2010). According to the standardized interview procedure, this assessment can be supported by clinical history from child health record, family statements, teachers’ evaluations, and comments on school reports [∼90 min].

The DIVA-5 was administered by a trained psychologist, while comorbidity assessments (MINI-S and DDSI) were performed by a trained psychiatrist or psychologist, with at least one done by a psychiatrist. The three interviews followed a set order: MINI-S first, DDSI second, and DIVA-5 last. Each assessor was blind to the results of the preceding assessments, except the DIVA-5 assessor, who was informed of psychiatric comorbidities identified by the MINI-S and DDSI, but was blind to specific ADHD modules of both comorbidities assessments.

**Fig. 1:**
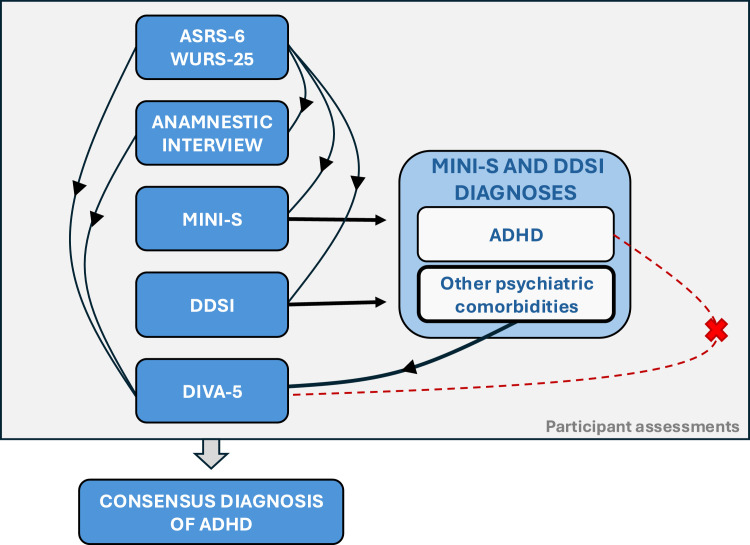
Full assessment procedure leading to Consensus Diagnosis of ADHD, highlighting how certain assessments were blinded from certain others. Description: The assessments are presented in the order in which they were carried out, from top to bottom. Black arrows represent information transmission; thus, the absence of arrows shows where assessments were blind from each other. The additional dotted red line further shows that DIVA assessment was blinded to the ADHD diagnosis evaluated by the MINI-S and DDSI, but not to their other psychiatric data.

At the end of the final assessment, all the assessors and the clinician supervisors meet to discuss the results of each assessment and to reach a consensus on ascertaining or refuting the diagnosis of ADHD, considering all the information collected. Clinicians followed a standardized procedure for conducting and scoring assessments but were encouraged to extend the assessment with unstructured interview if needed, to improve the final diagnosis consensus. For the Consensus Diagnosis, self-reported questionnaires are presented first, followed by conclusions of MINI-S and DDSI assessors. Finally, the conclusions of the DIVA-5 assessor are presented and all assessors discuss the ADHD diagnosis and differential diagnosis, regarding the output of all assessments, for the Consensus Diagnosis. The screening and diagnostic tools were all rated independently before the Consensus Diagnostic discussion began (Fig. 1).

We used the terms ‘Consensus Diagnosis’ when ADHD was ascertained using the full procedure, and ‘DIVA ADHD diagnosis’, ‘MINI-S ADHD diagnosis’ or ‘DDSI ADHD diagnosis’ for ADHD diagnosis based on the corresponding interviews. We report SUDs as categorized by the MINI-S, which mainly differentiates AUD from other SUDs.

Eighty-two patients were consecutively recruited between February 2021 and March 2024 (∼one every week), and included in the current analysis.

### Statistical analysis

For the current study, we report the results from anamnestic interview, self-report ADHD screening tools and structured interviews for ADHD diagnosis.

First, descriptive statistics were computed for the whole sample and as a function on the ADHD Consensus Diagnosis. Group comparisons were performed using the Mann-Whitney test for continuous variables and the Pearson’s χ^2^ or the Fisher exact test for nominal variables, depending on assumption checks.

Second, the Consensus Diagnosis was used as the valid criterion for the calculation of Sensitivity, Specificity, Positive Predictive Value (PPV) and Negative Predictive Value (NPV). Given the known dependence of predictive values on disease prevalence, PPV and NPV were further estimated across a range of hypothetical ADHD prevalence values, including 23 % as the reference for the prevalence of ADHD in SUD samples (van Emmerik-van Oortmerssen et al., 2012). This approach allows clinicians to interpret test performance according to the assumed prevalence in their specific setting. Predictive values were calculated using standard formulas: PPV = [Se × Prev] / [(Se × Prev) + ((1 − Sp) × (1 − Prev))] and NPV = [Sp × (1 − Prev)] / [((1 − Se) × Prev) + (Sp × (1 − Prev))].

The ASRS-6 has been presented in two scoring methodologies: (1) the sum score of dichotomized items, with a cut-off score of ≥4 for ADHD, as proposed in Kessler et al. (2005); and (2) the total score of all items, scored on a 0–4 likert scale from “never” to “very often”. ASRS diagnosis accuracy has been calculated with the first scoring methodology. The WURS-25 was first analysed using the standard scoring procedure (« WURS-25 sum scores »), defined as the unweighted sum of the 25 items, with a cut-off score of ≥46 for ADHD (Brevik et al., 2020). Second, fitted values (« WURS-25 fitted values ») were computed based on the 3-factor structure using the logistic regression coefficients proposed by Reimherr et al. (2022).

All computations were performed using R-Studio (V. 2023.12.0 + 369).

## Results

### Sample description

Participants were 74 % men, median age =36 years old (InterQuartile Range, IQR = 28–43) and 29 % were in a relationship (21 % married or living with a partner). Most (84 %) had a high school degree or higher, 41 % of whom had a master’s degree or higher. Fifty-eight percent were unemployed at the inclusion, including one retired, seven (15 %) on sick leave and 10 (22 %) students.

The most commonly reported primary SUD at referral was stimulant use disorder, accounting for 48 % of the total sample (24 % for synthetic cathinones, 22 % for cocaine, and 1 % for methamphetamine). Other main SUDs were alcolol and cannabis (23 % each). As regards past-year substance use, alcohol was the most commonly reported substance (84 %), followed by tobacco (66 %), cannabis (55 %), cocaine (47 %) and opioids (10 %) (Table 1). According to the MINI-S, 32 % had current AUD (69 % severe) and 61 % other SUDs (86 % severe). Twenty-five percent had both current AUD and SUD. Among those with no AUD or no SUD, 32 % reported a past AUD (with a 50 % distribution between prolonged and early remission) and 26 % reported a past SUD (with 62 % in early remission and 38 % in sustained remission) (Supplementary Table 1).

**Table 1:** Socio-demographic and clinical variables, as function of ADHD Consensus Diagnosis.

Variables	N	No ADHD, *N* = 33	ADHD, *N* = 49	p-value	Total (*N* = 82)
Age	82	39 (34, 45)	34 (27, 42)	**0.033**	36 (28, 43)
BMI	82	23.2 (20.4 - 26.2)	22.9 (19.8 - 24.9)	0.5	23 (20.3 - 25.6)
Gender	82			0.2	
Female		6 (18 %)	15 (31 %)		21 (26 %)
Male		27 (82 %)	34 (69 %)		61 (74 %)
Single	82	21 (64 %)	37 (76 %)	0.2	58 (71 %)
High school degree or more	82	26 (79 %)	43 (88 %)	0.3	69 (84 %)
Currently not working	82	17 (53 %)	29 (60 %)	0.5	46 (58 %)
**Main SUD at referral**	82			0.3	
Alcohol		8 (24 %)	11 (22 %)		19 (23 %)
Cannabis		5 (15 %)	14 (29 %)		19 (23 %)
Opiates		2 (6.1 %)	0 (0 %)		2 (2.4 %)
Others		1 (3.0 %)	2 (4.1 %)		3 (3.7 %)
Stimulant		17 (52 %)	22 (45 %)		39 (48 %)
Main SUD DSM-5 criteria	82	7 (5 - 8)	8 (7 - 9)	0.089	8 (6 - 9)
**Current substance use**					
Tobacco	82	23 (70 %)	31 (63 %)	0.5	54 (66 %)
Alcohol	81	30 (94 %)	38 (78 %)	0.052	68 (84 %)
Standard alcohol unit / drinking day	60	4 (2 - 8)	6 (3 - 10)	0.2	6 (2 - 9)
Cannabis	82	18 (55 %)	27 (55 %)	>0.9	45 (55 %)
Cocaine	81	17 (52 %)	21 (44 %)	0.5	38 (47 %)
Opioids	79	3 (9.1 %)	5 (11 %)	>0.9	8 (10 %)
**Psychiatric comorbidities**					
Any mood disorder	82	24 (73 %)	40 (82 %)	0.3	64 (78 %)
Any anxiety disorder	82	25 (76 %)	36 (73 %)	0.8	61 (74 %)
Lifetime suicide attempt	82	12 (36 %)	19 (39 %)	0.8	31 (38 %)
Number of DSM-5 disorders	82	4 (3 - 5)	4 (3 - 5)	>0.9	4 (3 - 5)
Any current psychiatric comorbidity	82	29 (88 %)	45 (92 %)	0.7	74 (90 %)

Data are presented as median (interquartile range) or n ( %). Group comparisons: Wilcoxon-Mann-Whitney test, Pearson’s chi-square, Fisher exact test. Psychiatric comorbidities were measured by MINI-S. BMI, Body Mass Index.

Fifty-seven (71 %) participants presented significant ADHD symptoms during childhood (above the WURS-25 cut-off score) and seventy (88 %) during adulthood (above the ASRS-6 cut-off score) (Table 2).

**Table 2:** ADHD variables, as function of ADHD Consensus Diagnosis.

Variables	N	No ADHD, *N* = 33	ADHD, *N* = 49	p-value	Total (*N* = 82)
WURS-25 total score	80	47 (37, 67)	61 (52, 67)	**0.046**	58 (40, 67)
WURS-25 above cut off (sum scores)	80	18 (56 %)	39 (81 %)	**0.016**	57 (71 %)
WURS-25 above cut off (fitted values)	80	14 (44 %)	31 (65 %)	0.066	45 (56 %)
ASRS-6 total score (dichotomized)	80	5 (4.75 - 6)	5.00 (5 - 6)	0.8	5 (5 - 6)
ASRS-6 total score	80	19 (15.8 - 20.3)	19 (17.8 - 21.3)	0.4	19 (17 - 21)
ASRS-6 above cut off	80	26 (81 %)	44 (92 %)	0.2	70 (88 %)
Current ADHD criteria	82	6 (4 - 9)	13 (11 - 14)	**<0.001**	11 (7.3 - 13)
ADHD criteria during childhood	82	5 (3 - 7)	11 (7.0 - 13)	**<0.001**	7.0 (5 - 12)

Data are presented as median (interquartile range) or n (%). Group comparisons: Wilcoxon-Mann-Whitney test, Pearson’s chi-square, Fisher exact test. Current and childhood ADHD criteria were measured by DIVA-5. ADHD, Attention Deficit Hyperactivity Disorder; WURS-25, Wender Utah Rating Scale, 25 items – presented using two scoring methods: (1) traditional sum scores and (2) fitted values computed using a logistic regression model based on the instrument’s 3-factor structure; ASRS, Adult Self Report Scale for ADHD, 6 items.

#### Psychiatric comorbidity

Seventy-four (90 %) participants suffered from at least one current psychiatric comorbidity according to the MINI-S (excluding SUD and ADHD), especially major depressive episode (54 %) and 24 % bipolar disorder (80 % type 1). Generalized anxiety disorder (GAD) was the most commonly reported anxiety disorder with 43 % in the total sample. Bulimia and anorexia nervosa were found in 9 % and 2 % of the population, respectively (Supplementary Table 1).

### Alignment of structured interviews with the consensus diagnosis for ADHD

Forty-nine participants (60 %) received a final Consensus Diagnosis of ADHD (Supplementary Table 3). ADHD participants were significantly younger than non-ADHD participants (Mann-Whitney, *U* = 1034.5, *p* = 0.033).

No significant differences were observed between participant who received a Consensus Diagnosis of ADHD compared to those who did not with regards to psychiatric comorbidity assessed using the MINI-S or the DDSI (Supplementary Tables 1–2).

#### DIVA-5 vs. consensus diagnosis

According to the DIVA-5, 52 participants (63 %) suffered from ADHD (Supplementary Table 3). In adulthood, participants had a median of six inattention criteria (IQR = 3–7), five hyperactive-impulsive criteria (IQR = 3–6) and reported impaired functioning in three domains (IQR = 2–4). During childhood, they had a median of four inattention criteria (IQR = 2–6), four hyperactivity-impulsivity criteria (IQR = 2–7) and reported impaired functioning in two domains (IQR = 1–3) (Supplementary Table 5).

**Fig. 2:**
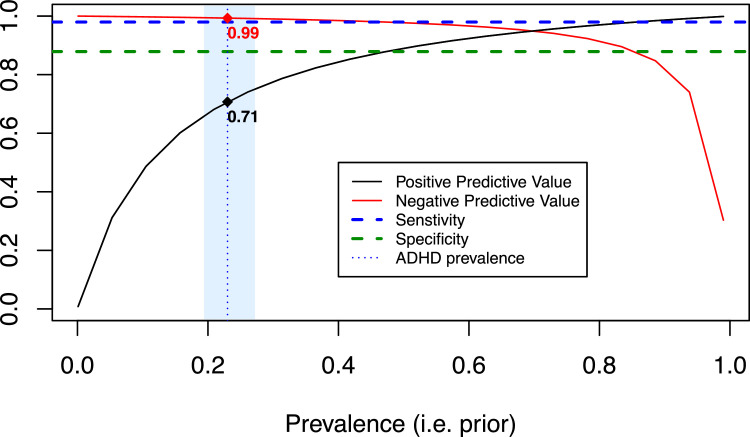
Diagnosis accuracy of the DIVA-5 for ADHD, using the Consensus Diagnostic as the validity criterion. Description: ADHD prevalence corresponds to the assumed ADHD prevalence in the sample, 23 %, including confidence interval represented by the blue area.

The DIVA-5 conclusions did not align with the Consensus ADHD Diagnosis in 6 % cases. Using the Consensus Diagnosis as reference, the DIVA-5 showed a sensitivity of 0.98, a specificity of 0.88, PPV of 0.92 and a NPV of 0.97. When tested with an assumed prevalence of ADHD of 23 % in the SUD sample, the PPV decreased to 0.71 and the NPV increased to 0.99 (Table 3). Analyses on various ADHD prevalence showed that the DIVA-5 NPV remained high for a prevalence of ADHD up to 85 %. The DIVA-5 PPV increased as the simulated prevalence increased (Fig. 2).

**Table 3:** Psychometric properties of screening and diagnostic tools for adult ADHD compared with the Consensus Diagnosis as the external criterion.

	Sensitivity	Specificity	PPV	PPV-23 %	NPV	NPV-23 %
**MINI-S**	0.88	0.30	0.65	0.27	0.62	0.89
**DDSI**	0.84	0.48	0.71	0.33	0.67	0.91
**DIVA-5**	0.98	0.88	0.92	0.71	0.97	0.99
**ASRS-6**	0.92	0.19	0.63	0.25	0.60	0.88
**WURS-25 (sum scores)**	0.81	0.44	0.68	0.30	0.61	0.89
**WURS-25 (fitted values)**	0.65	0.56	0.69	0.28	0.51	0.86

MINI-S, Mini International Neuropsychiatric Interview Simplified; DDSI, Dual Disorder Screening Interview; DIVA-5, Diagnostic Interview for ADHD in adults; ASRS-6, Adult ADHD Self-Report Scale – 6 items; WURS-25, Wender Utah Rating Scale, 25 items – presented using two scoring methods: (1) traditional sum scores and (2) fitted values computed using a logistic regression model based on the instrument’s 3-factor structure; PPV, Positive Predictive Value; NPV, Negative Predictive Value; PPV-23 % and NPV-23 %, PPV and NPV calculated for an assumed prevalence of 23 % for ADHD in the sample.

#### MINI-S vs. consensus diagnosis

Sixty-six participants (80 %) were diagnosed with ADHD according to the MINI-S. Eighty-eight percent of ascertained ADHD patients also screened positive for ADHD with the MINI-S (Supplementary Table 1).

The MINI-S did not align with the Consensus ADHD Diagnosis in 35 % cases. Using the Consensus Diagnosis as reference, the MINI-S had a sensitivity of 0.88, a specificity of 0.3, a PPV of 0.65 and a NPV of 0.62. When adjusted for the estimated prevalence of ADHD in the SUD sample (23 %), the PPV decreased to 0.27 and the NPV increased to 0.89 (Table 3). The graph of PPV and NPV depending on assumed prevalence of ADHD in the sample is available as Supplementary Figure 1.

#### DDSI vs. consensus diagnosis

ADHD was the most frequent diagnosis according to the DDSI (*n* = 58, 71 %), followed by MDD, which was identified in 59 % of the sample. The third most common diagnosis was PTSD (28 %). Lifetime mania was identified in 5 % individuals by the DDSI (Supplementary Table 2).

The DDSI did not align with the ADHD Consensus Diagnosis in 30.5 % cases. Using the Consensus Diagnosis as reference, the DDSI had a sensitivity of 0.84, a specificity of 0.48, a PPV of 0.71 and a NPV of 0.67. When tested with an assmued prevalence of 23 % ADHD in a SUD sample, the PPV decreased to 0.33 and the NPV increased to 0.91 (Table 3). The graph of PPV and NPV depending on assumed prevalence of ADHD in the sample is available as Supplementary Figure 2.

### Alignment of self-rated scales with the consensus diagnosis for ADHD

#### WURS-25 vs. consensus diagnosis

The median WURS-25 score was 58 (IQR = 40–67). Based on the cut-off of 46, 71 % of the total sample had probable child ADHD, according to the classical scoring method, and 56 % with the fitted values scoring (Table 2). The WURS-25 did not align with the Consensus Diagnosis of ADHD in 34–39 % of cases (Supplementary Table 4). Compared to the Consensus Diagnosis of ADHD, the WURS-25 (sum scores) had a sensitivity of 0.81, a specificity of 0.44, a PPV of 0.68 and a NPV of 0.61 (respectively 0.65, 0.56, 0.69, 0.51 for the fitted values scoring). When adjusted for an assumed prevalence of ADHD in the SUD population of 23 %, the PPV decreased to 0.28–0.3 and the NPV increased to 0.86–0.89 (Table 3). The graph of PPV and NPV depending on assumed prevalence of ADHD in the sample is available as Supplementary Figure 3–4.

#### ASRS-6 vs. consensus diagnosis

The median ASRS-6 score was five (IQR = 5–6) for the dichotomized scoring and 19 (IQR = 17–21) for the continuous scoring. Eighty-eight percent of the total sample scored above the cut-off score (cut-off > 3) (Table 2). The ASRS-6 did not align with the Consensus Diagnosis of ADHD in 37 % of cases (Supplementary Table 4). Compared to the Consensus Diagnosis of ADHD, the ASRS-6 had a sensitivity of 0.92, a specificity of 0.19, a PPV of 0.63 and a NPV of 0.6. When adjusted to an assumed prevalence of ADHD =23 % in SUD samples, the PPV decreased to 0.25 and the NPV increased to 0.88 (Table 3). The graph of PPV and NPV depending on assumed prevalence of ADHD in the sample is available as Supplementary Figure 5.

## Discussion

In the current study, a multi-disciplinary and multi-tools procedure allowed clinicians to reach consensus for complex ADHD diagnosis in an SUD population. In the sample, SUDs were typically multiple, severe and/or comorbid with other psychiatric disorders. The main finding was that all five instruments showed good to excellent sensitivity (0.84 - 0.98) when compared to the Consensus Diagnosis, whereas only the DIVA-5 showed good specificity (0.88). Thus, for the four other tools, specificity was poor to very poor (0.19 – 0.48). Importantly, both the PPV and NPV for an assumed prevalence of ADHD in the sample of 23 % were high only for the DIVA-5 (PPV = 0.71, NPV = 0.99).

To the best of our knowledge, our study adds precious knowledge to previous literature regarding the usefulness of the DIVA-5 compared to other diagnostic instruments for diagnosing ADHD in an SUD sample with high rates of addictive and psychiatric comorbidities. With that regard, we found no difference between participants who were diagnosed with ADHD, and those who were not - except regarding their younger age. Importantly, the assessments were all carried out in the same sample, allowing the results of the instruments to be compared without between-sample effects. The sample was relatively large, given the clinical complexity of the patients, and was extensively characterized by standard instruments.

Only the DIVA-5 showed good diagnostic accuracy in our sample, supporting our initial hypothesis. Importantly, the PPV and NPV of the DIVA-5 remained stable and high for several estimates of ADHD prevalence. Conversely, the MINI-S, the DDSI, the ASRS-6 and the WURS-25 carried a high risk of false-positives, with a maximum PPV = 33 %. This is encouraging as regards the use of the DIVA-5 in samples with a high assumed prevalence of ADHD, such as those with SUD or prison inmates. Conversely, this should be considered to limit the use of the other instruments tested in the study to screening procedures, especially since the MINI-S is often regarded as diagnostic tool. However, the NPV of the WURS-25 and ASRS-6 were similar to that of the MINI-S and the DDSI, whereas WURS-25 and ASRS-6 are only self-reported questionnaires. Since the former require much less time and training than the latter, clinicians may consider using the MINI-S or the DDSI only to investigate a whole set of psychiatric comorbidities - provided they were trained to do so. The findings also remind that the ASRS-6 and the WURS-25 have been designed for screening purposes only, and that they should not be used for diagnostic ascertainment, even more so in the context of complex psychiatric comorbidity. Overall, our findings support the reliability of using ADHD screening tools in SUD populations, as previously evidenced (Crunelle et al., 2018; Palma-Álvarez et al., 2020). Previous studies of DSM-IV version of the MINI-S, the MINI-Plus, and of the DDSI also investigated their diagnosis accuracy for ADHD, in comparison with the CAADID (Mestre-Pintó et al., 2013; Palma-Álvarez et al., 2020). They report similar results as ours in terms of sensitivity of the two instruments, while the DDSI showed a higher specificity =85 %. In the DDSI study, the PPV was also acceptable (68 %) and comparable to ours (71 %). However, this study did not include a recalculation of the Se and Sp for a simulated prevalence of ADHD = 23 %, which is assumed in SUD samples. By doing so, we showed that the PPV of the DDSI was strongly reduced, discouraging its use as a diagnostic interview for ADHD in SUDs patients.

To the best of our knowledge, only one study describing the validity of the DIVA 2.0 (DSM-IV version)(Luderer et al., 2020) in people with SUDs had been published to date. This study showed a moderate correlation between the DIVA 2.0 and the external criteria (expert diagnosis) (Kappa = 0.56), while the DIVA-5 correctly classified 94 % of our sample regarding ADHD. However, the DIVA 2.0 study fairly differ in terms of clinical severity and diversity of the participants, which were limited to abstinent patients in residential care for alcohol use disorder, and the procedure was both highly conservative and sensitive to the effects of the repeated assessments and of alcohol abstinence.

There were some limitations to this study. Firstly, the recruitment process only selected participants who were suspected of having ADHD by their referring clinician. Therefore, the whole sample had significant ADHD symptoms, reducing the likelihood of false negatives. Also, recent substance use has been reported for the past 12 months and does not allow testing the effect of very recent substance use on instrument performance. Moreover, the study design focused only on the short forms of ADHD screening questionnaires, and further research is needed to assess the diagnostic accuracy of other versions of the ASRS and WURS in this specific clinical population. Another limitation concerns the lack of blinding of the external criteria for ADHD assessments. Finally, a major limitation relates to the DIVA-5 assessment methodology. Although the DIVA-5 assessor was blinded to the ADHD results of the other interviews, the comorbidity results were accessible to the DIVA-5 assessor. Therefore, the DIVA-5 psychometric results presented in this article rather refers to a combination of MINI-S + DDSI (excluding the ADHD modules) + DIVA-5 than to the DIVA-5 alone. It is therefore realistic to hypothesize that the DIVA-5 assessor's knowledge of psychiatric comorbidities and symptoms may lead to good DIVA-5 performance in differential diagnosis.

As the recruitment procedure included participants with a high suspicion of ADHD, the generalizability of the results may be limited, especially regarding the usefulness of screening assessments in other populations. Moreover, in our study population, 90 % of the sample suffered from at least one comorbid psychiatric disorder (excluding SUD and ADHD), which is much higher that the prevalence found in another sample of adults with ADHD+SUD (Brynte et al., 2022). Moreover, the SUDs appeared severe in our sample with 75 % of the population who presented six or more SUD symptoms and the most represented primary substance of abuse was cocaine whereas alcohol was more prevalent in other studies (Brynte et al., 2022; van de Glind et al., 2014).

## Conclusion

This study reports on the diagnostic accuracy of screening and diagnostic instruments for ADHD in adults who suffer from a SUD. While all instruments showed good sensitivity, only the DIVA-5 showed both good sensitivity and specificity, compared to an expert and multi-disciplinary Consensus Diagnosis. The substantial risk of false positives on the two self-reported questionnaires (ASRS-6, WURS-25), and the two structured interviews (MINI-S and DDSI) suggests that significant ADHD symptoms reported on these instruments should not lead to a diagnosis of ADHD but require further investigation. Our findings strongly suggest that the DIVA-5 represents a reliable basis for such investigation.

## Ethics

The study was conducted according to the tenets of the Declaration of Helsinki (World Medical Association, 2013) and the rules of the ethics committee of Paris-Nanterre University. In accordance with standard care at Paris academic hospitals, participants were informed that their data may be used for research, with the option to object. All participants signed a consent form stating they did not object to this use. No additional approval was required.

## Access to data

NT and RI had full access to data and take responsibility for the integrity of the data and the accuracy of the data analysis. Data is unavailable to access because of it includes sensitive/confidential information (patient data).

## Funding source

This research did not receive any specific grant from funding agencies in the public, commercial, or not-for-profit sectors.

## Declaration of competing interest

NT, FV, EK, RI receive royalties from ELSEVIER MASSON S.A.S. for the French translation of the following book: Matthys et al. (2018). *Managing ADHD in the presence of substance use disorders*. Gompel & Svacina. Other authors declare the absence of any commercial or financial relationship that could be construed as a potential conflict of interest.

## Appendix Supplementary materials

Image, application 1
