# Supplementary material for: Psychometric properties of ADHD screening and diagnostic tools in patients with severe substance use disorders
Source: Int J Clin Health Psychol. 2026 Apr 16;26(1):100682. doi: 10.1016/j.ijchp.2026.100682 (PMC13099338; doi:10.1016/j.ijchp.2026.100682)
Supplement: Supplementary file 1 [file mmc1.docx]

**Psychometric Properties of ADHD Screening and Diagnostic Tools in Patients with Severe Substance Use Disorders.**

**SUPPLEMENTARY MATERIAL**

[1. Supplementary Tables 2](#_Toc226109427)

[1.1. Supplementary Table 1: MINI-S diagnosis, as function of ADHD Consensus Diagnosis 2](#_Toc226109428)

[1.2. Supplementary Table 2: DDSI diagnosis, as function of ADHD Consensus Diagnosis 2](#_Toc226109429)

[1.3. Supplementary Table 3: Contingency table for structures diagnosis interviews, as function of ADHD Consensus Diagnosis 3](#_Toc226109430)

[1.4. Supplementary Table 4: Contingency table for screening tools for ADHD as function of ADHD Consensus Diagnosis 3](#_Toc226109431)

[1.5. Supplementary Table 5: DIVA-5 results, as function of ADHD Consensus Diagnosis 3](#_Toc226109432)

[2. Supplementary Figures 4](#_Toc226109433)

[2.1. Supplementary Figure 1: Diagnosis accuracy of the MINI-S for ADHD, using the Consensus Diagnostic as the external criterion 4](#_Toc226109434)

[2.2. Supplementary Figure 2: Diagnosis accuracy of the DDSI for ADHD, using the Consensus Diagnostic as the external criterion 4](#_Toc226109435)

[2.3. Supplementary Figure 3: Diagnosis accuracy of the WURS-25 (sum scores) for ADHD, using the Consensus Diagnostic as the external criterion 5](#_Toc226109436)

[2.4. Supplementary Figure 4: Diagnosis accuracy of the WURS-25 (fitted values) for ADHD, using the Consensus Diagnostic as the external criterion 5](#_Toc226109437)

[2.5. Supplementary Figure ~~4~~ 5: Diagnosis accuracy of the ASRS-6 for ADHD, using the Consensus Diagnostic as the external criterion. 6](#_Toc226109438)

# Supplementary Tables

## Supplementary Table 1: MINI-S diagnosis, as function of ADHD Consensus Diagnosis

| **Variables** | **N** | **No ADHD**  N = 33 | **ADHD**  N = 49 | **p-value** | **Total**  N = 82 |
| --- | --- | --- | --- | --- | --- |
| Major Depressive Disorder | 44 |  |  | 0.8 |  |
| Recurrent |  | 11 (33.3%) | 18 (36.7%) |  | 29 (35.4%) |
| Single |  | 7 (21.2%) | 8 (16.3%) |  | 15 (18.3%) |
| Bipolar Disorder | 20 |  |  | >0.9 |  |
| BD I |  | 5 (15.2%) | 11 (22.4%) |  | 16 (19.5%) |
| BD II |  | 1 (3%) | 3 (6.1%) |  | 4 (4.9%) |
| Bulimia Nervosa | 82 | 3 (9.1%) | 4 (8.2%) | >0.9 | 7 (8.5%) |
| Anorexia Nervosa | 82 | 1 (3%) | 1 (2.0%) | >0.9 | 2 (2.4%) |
| Substance Use Disorder | 71 |  |  | 0.6 |  |
| Current |  | 19 (57.6%) | 31 (63.3%) |  | 50 (61%) |
| Early remission |  | 7 (21.2%) | 6 (12.2%) |  | 13 (15.9%) |
| Sustained remission |  | 3 (9.1%) | 5 (10.2%) |  | 8 (9.8%) |
| Alcohol Use Disorder | 52 |  |  | >0.9 |  |
| Current |  | 10 (30.3%) | 16 (32.7%) |  | 26 (31.7%) |
| Early remission |  | 5 (15.2%) | 8 (16.3%) |  | 13 (15.9%) |
| Sustained remission |  | 5 (15.2%) | 8 (16.4%) |  | 13 (15.9%) |
| Social Anxiety Disorder | 82 | 11 (33%) | 16 (33%) | >0.9 | 27 (33%) |
| Panic Disorder | 82 | 4 (12%) | 5 (10%) | >0.9 | 9 (11%) |
| Agoraphobia | 82 | 3 (9.1%) | 6 (12%) | 0.7 | 9 (11%) |
| Generalized Anxiety Disorder | 82 | 14 (42%) | 21 (43%) | >0.9 | 35 (43%) |
| Obsessive Compulsive Disorder | 82 | 3 (9.1%) | 10 (20%) | 0.2 | 13 (16%) |
| Post-Traumatic Stress Disorder | 82 | 10 (30%) | 9 (18%) | 0.2 | 19 (23%) |
| ADHD | 82 | 23 (70%) | 43 (88%) | **0.043** | 66 (80%) |
| Delusional Disorder | 82 | 0 (0%) | 0 (0%) |  | 0 (0%) |
| Brief Psychotic Disorder | 82 | 1 (3.0%) | 1 (2.0%) | >0.9 | 2 (2.4%) |
| Schizophreniform Disorder | 82 | 0 (0%) | 0 (0%) |  | 0 (0%) |
| Schizophrenia | 82 | 0 (0%) | 0 (0%) |  | 0 (0%) |

**Supplementary Table 1**: MINI-S diagnosis, as function of ADHD Consensus Diagnosis.

Data are presented as n (%). Group comparisons: Pearson’s chi-square or Fisher exact test.

## Supplementary Table 2: DDSI diagnosis, as function of ADHD Consensus Diagnosis

| **Variables** | **N** | **No ADHD**  N = 33 | **ADHD**  N = 49 | **p-value** | **Total**  N = 82 |
| --- | --- | --- | --- | --- | --- |
| Panic Disorder | 82 | 2 (6.1%) | 1 (2.0%) | 0.6 | 3 (3.7%) |
| Generalized Anxiety Disorder | 82 | 5 (15%) | 6 (12%) | 0.7 | 11 (13%) |
| Specific Phobia | 82 | 3 (9.1%) | 6 (12%) | 0.7 | 9 (11%) |
| Social Anxiety Disorder | 82 | 7 (21%) | 14 (29%) | 0.5 | 21 (26%) |
| Agoraphobia | 82 | 4 (12%) | 12 (24%) | 0.2 | 16 (20%) |
| Dysthymia | 82 | 9 (27%) | 12 (24%) | 0.8 | 21 (26%) |
| Major Depressive Disorder | 82 | 18 (55%) | 30 (61%) | 0.5 | 48 (59%) |
| Lifetime Mania | 82 | 0 (0%) | 4 (8.2%) | 0.14 | 4 (4.9%) |
| Suspected Psychosis | 82 | 0 (0%) | 3 (6.1%) | 0.3 | 3 (3.7%) |
| ADHD | 82 | 17 (52%) | 41 (84%) | **0.002** | 58 (71%) |
| Post-Traumatic Stress Disorder | 82 | 11 (33%) | 12 (24%) | 0.4 | 23 (28%) |

**Supplementary Table 2:** DDSI diagnosis, as function of ADHD Consensus Diagnosis.

Data are presented as n (%). Group comparisons: Pearson’s chi-square or Fisher exact test.

## Supplementary Table 3: Contingency table for structures diagnosis interviews, as function of ADHD Consensus Diagnosis

|  | **ADHD MINI-S** | | **ADHD DDSI** | | **ADHD DIVA-5** | | **Total** |
| --- | --- | --- | --- | --- | --- | --- | --- |
| **ADHD Consensus Diagnosis** | No | Yes | No | Yes | No | Yes |  |
| No | 10 | 23 | 16 | 17 | 29 | 4 | 33 |
| Yes | 6 | 43 | 8 | 41 | 1 | 48 | 49 |
| **Total** | **16** | **66** | **24** | **58** | **30** | **52** | **82** |

**Supplementary Table 3**: Contingency table for structured diagnosis interviews as function of ADHD Consensus Diagnosis. MINI-S, Mini International Neuropsychiatric Interview Simplified; DDSI, Dual Disorder Screening Instrument; DIVA-5, Diagnostic Interview for ADHD in adults.

## Supplementary Table 4: Contingency table for screening tools for ADHD as function of ADHD Consensus Diagnosis

|  | **ADHD WURS-25 (sum scores)** | | **ADHD WURS-25**  **(fitted values)** | | **ADHD ASRS-6** | |
| --- | --- | --- | --- | --- | --- | --- |
| **ADHD Consensus Diagnosis** | No | Yes | No | Yes | No | Yes |
| No | 14 | 18 | 18 | 14 | 6 | 26 |
| Yes | 9 | 39 | 17 | 31 | 4 | 44 |
| **Total** | **23** | **57** | **35** | **45** | **10** | **70** |

**Supplementary Table 4:** Contingency table for screening tools for ADHD as a function of ADHD Consensus Diagnosis. WURS-25 (Wender Utah Rating Scale, 25 items) is presented using two scoring methods: (1) traditional sum scores and (2) fitted values computed using a logistic regression model based on the instrument’s 3-factor structure; ASRS-6 (Adult Self-Report Scale for ADHD, 6 items).

## Supplementary Table 5: DIVA-5 results, as function of ADHD Consensus Diagnosis

| **DIVA-5** | **N** | **No ADHD**, N = 33 | **ADHD**, N = 49 | **p-value** | **Total** (N = 82) |
| --- | --- | --- | --- | --- | --- |
| **General** | | | | | |
| ADHD presentation | 52 |  |  | 0.5 |  |
| Combined |  | 3 (75%) | 33 (69%) |  | 36 (69%) |
| Hyperactive-impulsive |  | 1 (25%) | 6 (13%) |  | 7 (13%) |
| Inattentive |  | 0 (0%) | 9 (19%) |  | 9 (17%) |
| Family testimony | 81 | 14 (42%) | 19 (40%) | 0.8 | 33 (41%) |
| **Adulthood** | | | | | |
| Attention criteria | 82 | 3 (1 - 5) | 7 (6 - 8) | **<0.001** | 6 (3 - 7) |
| >4 | 82 | 12 (36%) | 42 (86%) | **<0.001** | 54 (66%) |
| Hyperactive-Impulsive criteria | 82 | 3 (2 - 4) | 6 (5 - 7) | **<0.001** | 5 (3 - 6) |
| >4 | 82 | 8 (24%) | 40 (82%) | **<0.001** | 48 (59%) |
| Number of altered domains | 80 | 2 (1 - 2.25) | 3 (2 - 4) | **<0.001** | 3 (2 - 4) |
| >1 | 80 | 21 (66%) | 47 (98%) | **<0.001** | 68 (85%) |
| **Childhood** | | | | | |
| Attention criteria | 82 | 2 (1 - 4) | 5 (4 - 7) | **<0.001** | 4 (2 - 6) |
| >4 | 82 | 5 (15%) | 32 (65%) | **<0.001** | 37 (45%) |
| Hyperactive-Impulsive criteria | 82 | 2 (1 - 3) | 5 (3 - 8) | **<0.001** | 4 (2 - 7) |
| >4 | 82 | 5 (15%) | 29 (59%) | **<0.001** | 34 (41%) |
| Attention/Hyperactivity-Impulsivity criteria > 2 | 82 | 25 (76%) | 49 (100%) | **<0.001** | 74 (90%) |
| Number of altered domains | 80 | 1 (0.75 - 2) | 3 (2 - 3) | **<0.001** | 2 (1 - 3) |
| >1 | 80 | 11 (34%) | 44 (92%) | **<0.001** | 55 (69%) |

**Supplementary Table 5:** DIVA-5 results, as function of ADHD Consensus Diagnosis.

Data are presented as median (interquartile range) or n (%). Group comparisons: Pearson’s chi-square or Fisher exact test.

# Supplementary Figures

## Supplementary Figure 1: Diagnosis accuracy of the MINI-S for ADHD, using the Consensus Diagnostic as the external criterion


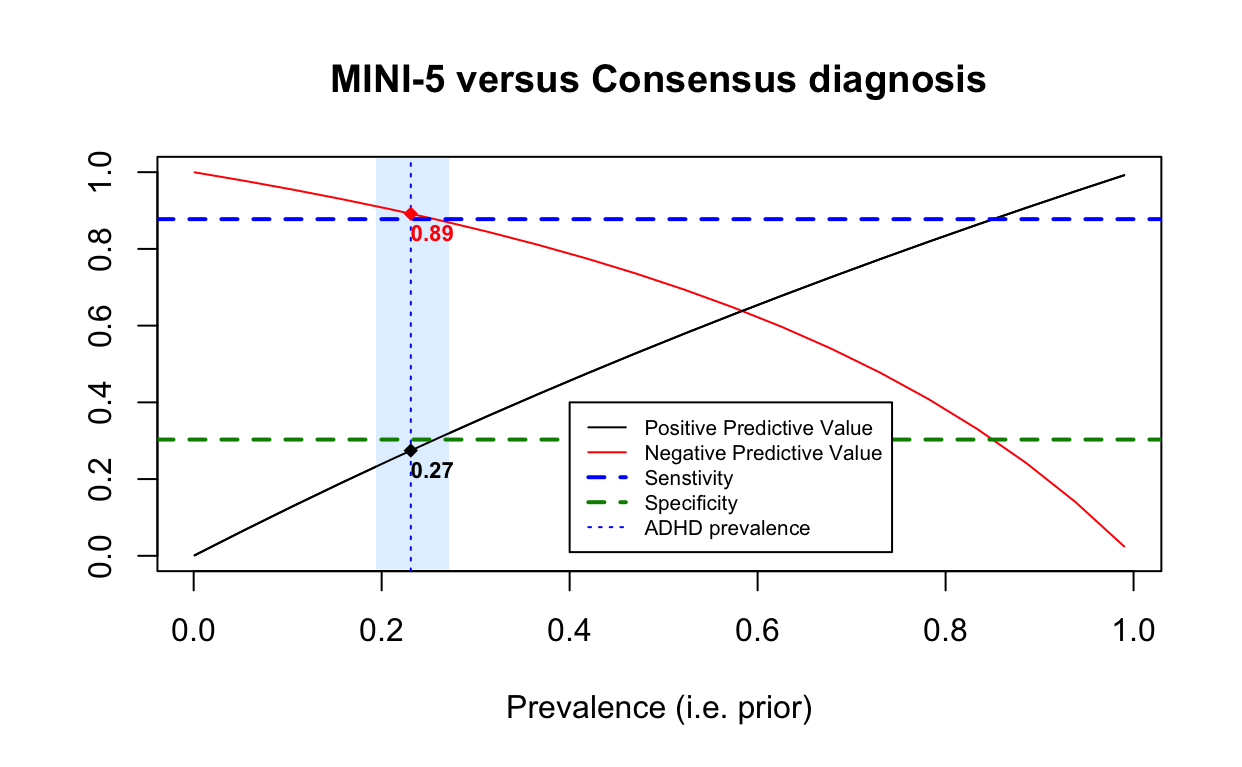


**Supplementary Figure 1:** Diagnosis accuracy of the MINI-S for ADHD, using the Consensus Diagnostic as the external criterion. ADHD prevalence corresponds to the expected ADHD prevalence in the sample, 23%, including confidence interval represented by the blue area.

## Supplementary Figure 2: Diagnosis accuracy of the DDSI for ADHD, using the Consensus Diagnostic as the external criterion


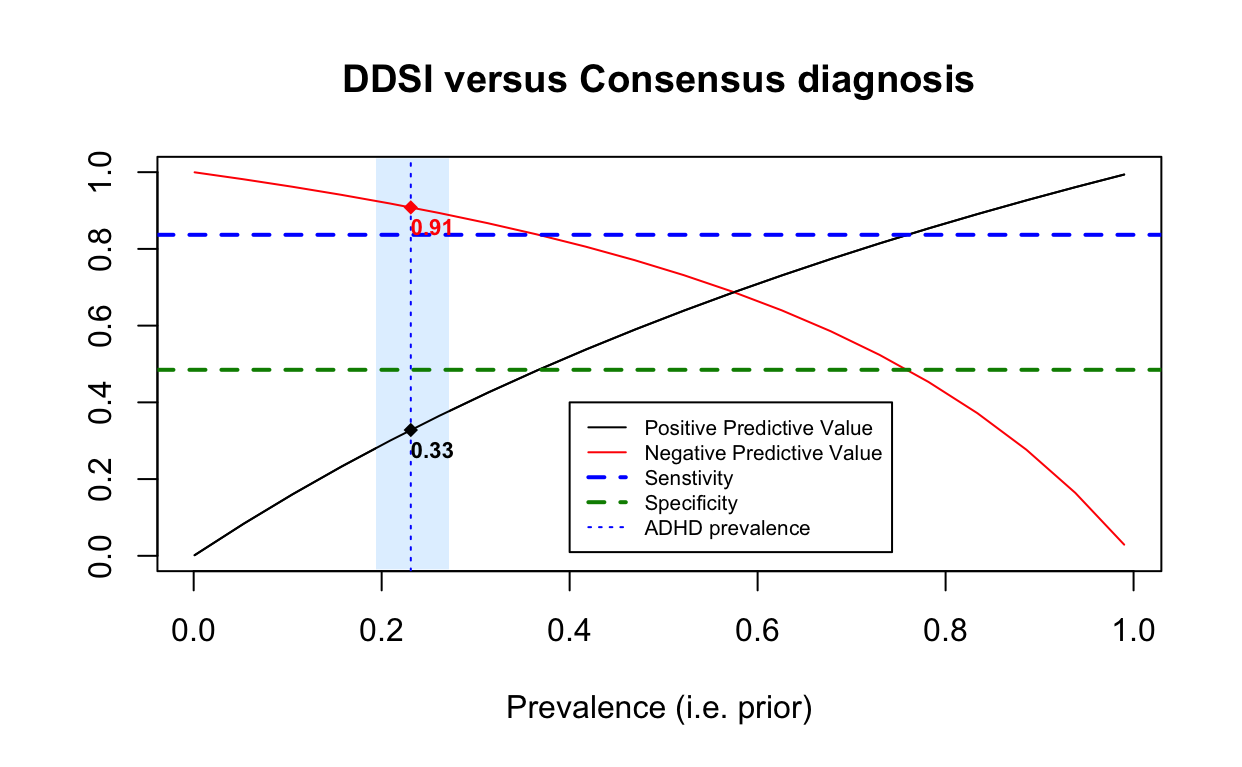


**Supplementary Figure 2:** Diagnosis accuracy of the DDSI for ADHD, using the Consensus Diagnostic as the external criterion. ADHD prevalence corresponds to the expected ADHD prevalence in the sample, 23%, including confidence interval represented by the blue area.

## Supplementary Figure 3: Diagnosis accuracy of the WURS-25 (sum scores) for ADHD, using the Consensus Diagnostic as the external criterion


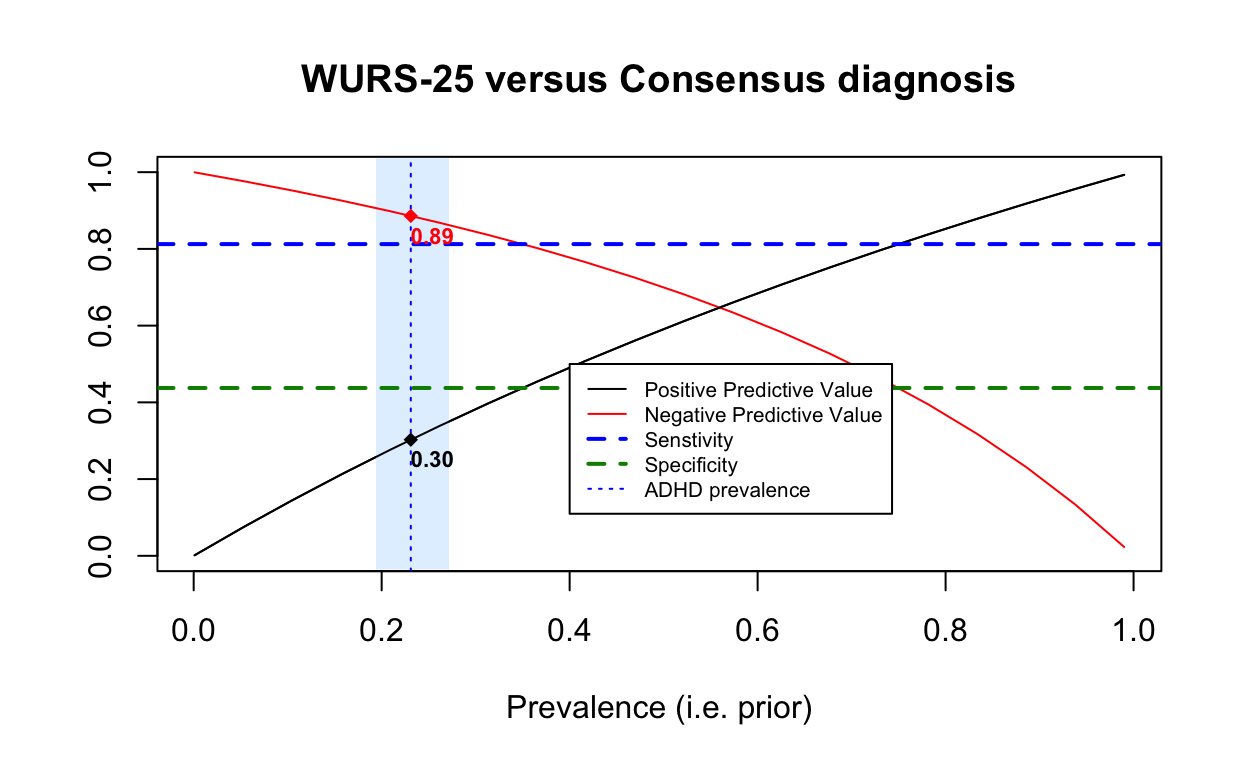


**Supplementary Figure 3:** Diagnosis accuracy of the WURS-25 (sum score) for ADHD, using the Consensus Diagnostic as the external criterion. ADHD prevalence corresponds to the expected ADHD prevalence in the sample, 23%, including confidence interval represented by the blue area.

## Supplementary Figure 4: Diagnosis accuracy of the WURS-25 (fitted values) for ADHD, using the Consensus Diagnostic as the external criterion


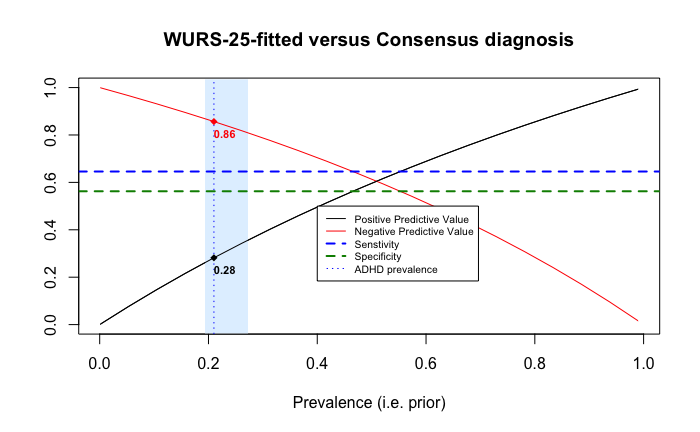


**Supplementary Figure 4:** Diagnosis accuracy of the WURS-25 (fitted values computed using a logistic regression model based on the instrument’s 3-factor structure) for ADHD, using the Consensus Diagnostic as the external criterion. ADHD prevalence corresponds to the expected ADHD prevalence in the sample, 23%, including confidence interval represented by the blue area.

## Supplementary Figure 5: Diagnosis accuracy of the ASRS-6 for ADHD, using the Consensus Diagnostic as the external criterion.


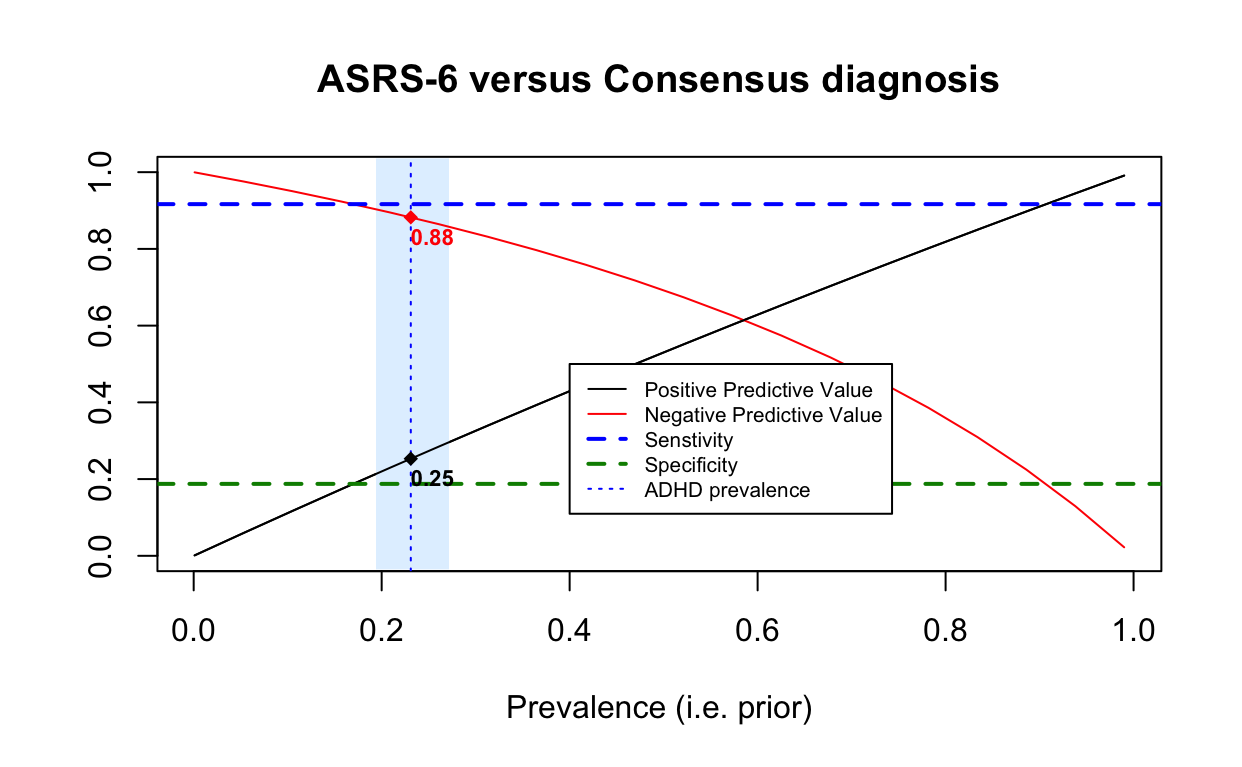


**Supplementary Figure 5:** Diagnosis accuracy of the ASRS-6 for ADHD, using the Consensus Diagnostic as the external criterion. ADHD prevalence corresponds to the expected ADHD prevalence in the sample, 23%, including confidence interval represented by the blue area
